# Trabalhadoras sexuais no marco pandêmico brasileiro: efeitos em e relações com a saúde

**DOI:** 10.1590/0102-311XPT181123

**Published:** 2024-09-20

**Authors:** Amanda de Mello Calabria, Nicolas Lorente, Michel de Oliveira Furquim dos Santos, Ana Carolina Braga Azevedo, Paula Galdino Cardin de Carvalho, Daniel Dutra de Barros, Gizelle Aparecida Oliveira, Océane Apffel Font, Silvana de Souza Nascimento, Maria Amelia de Sousa Mascena Veras, Daniela Rojas Castro, José Miguel Nieto Olivar

**Affiliations:** 1 Instituto de História, Universidade Federal Fluminense, Niterói, Brasil.; 2 Laboratoire de Recherche Communautaire, Coalition PLUS, Pantin, France.; 3 Centre d’Estudis Epidemiològics sobre les Infeccions de Transmissió Sexual i Sida de Catalunya, Departament de Salut, Badalona, España.; 4 Faculdade de Saúde Pública, Universidade de São Paulo, São Paulo, Brasil.; 5 Faculdade de Filosofia, Letras e Ciências Humanas, Universidade de São Paulo, São Paulo, Brasil.; 6 Faculdade de Ciências Médicas da Santa Casa de São Paulo, São Paulo, Brasil.; 7 Institut des Sciences de la Santé Publique, Aix Marseille Université, Marseille, France.

**Keywords:** Pandemia por COVID-19, Redes Comunitárias, Profissionais do Sexo, Ativismo Social, Cuidado Humanizado, COVID-19 Pandemics, Community Networks, Sex Workers, Social Activism, Humanization of Care, Pandemia de COVID-19, Redes Comunitarias, Trabajadores Sexuales, Activismo Social, Humanización de la Atención

## Abstract

Este trabalho apresenta os resultados do estudo *Eu Quero é Mais! A Vida de Profissionais do Sexo Durante a Pandemia da COVID-19*, integrante do programa de investigação comunitária EPIC. O estudo analisou os efeitos da pandemia nas vidas de trabalhadoras sexuais cis, trans e travestis em nove Unidades da Federação e 11 cidades brasileiras ao longo de 2020 e 2021. O artigo tem como foco o componente qualitativo do estudo baseado em entrevistas semiestruturadas realizadas de forma presencial e remota com 43 trabalhadoras sexuais, e seu cotejamento com o componente quantitativo. Os efeitos são analisados em relação com o marco pandêmico brasileiro, considerando as dimensões sociais, econômicas e políticas do vírus da COVID-19. Entre as temáticas chaves da análise, se destacam: casos de adoecimento, práticas localizadas de isolamento social, práticas de prevenção e gerenciamento de cuidado, vacinação individual e estratégias coletivas de vacinação. Compartilhamos também as respostas cotidianas e ativistas traçadas por trabalhadoras sexuais numa agenda política que se contrapõe à lógica individualista, familiarista, doméstica e neoliberal de isolamento, por meio de perspectivas comunitárias de cuidado, o que se desenhou como a única linha de ação em saúde para a categoria durante a pandemia. As ações coletivas reposicionam o trabalho sexual na interface entre a saúde pública e os direitos humanos e tomam como princípio os conhecimentos das ruas, desde os ativismos, e o poder de decisão delas próprias sobre seus corpos.

## Abstract (en)

This paper describes the results of the study *I Want More! The Lives of Sex Workers During the COVID-19 Pandemic*, which is part of the EPIC community research program. The study analyzed the effects of the pandemic on the lives of cis, trans and *travesti* sex workers in nine Brazilian states and 11 cities throughout 2020 and 2021. This article focuses on the qualitative component of the study, which was based on semi-structured, remote and face-to-face interviews carried out with 43 sex workers, and its comparison with the quantitative component. The effects are analyzed in relation to the Brazilian pandemic framework, considering the social, economic and political dimensions of the COVID-19 virus. Some of the key themes of the analysis are cases of illness, specific social isolation practices, prevention and care management practices, individual vaccination and collective vaccination strategies. We also share the daily and activist responses drawn up by sex workers in a political agenda that opposes the individualistic, familialist, domestic, and neoliberal logic of isolation by adopting community care perspectives, which was the only line of health action for this work category during the pandemic. Collective actions reposition sex work at the interface between public health and human rights and take as their principle the “street knowledge”, from activism, and the workers’ power of decision over their own bodies.

## Resumen

Este trabajo presenta los resultados del estudio *¡Yo Quiero Más! La Vida de las Profesionales del Sexo Durante la Pandemia de la COVID-19*, parte del programa de investigación comunitaria EPIC. El estudio analizó los efectos de la pandemia en la vida de trabajadoras sexuales cis, trans y *travestis* en 9 estados y 11 ciudades brasileñas a lo largo del 2020 y del 2021. El artículo se centra en el componente cualitativo del estudio basado en entrevistas semiestructuradas realizadas de forma presencial y remota a 43 trabajadoras sexuales y su comparación con el componente cuantitativo. Los efectos se analizan con relación al marco pandémico brasileño, considerando las dimensiones sociales, económicas y políticas del virus de la COVID-19. Entre las temáticas clave del análisis, destacan: casos de enfermedad, prácticas localizadas de aislamiento social, prácticas de prevención y gestión del cuidado, vacunación individual y estrategias colectivas de vacunación. Compartimos también las respuestas cotidianas y activistas esbozadas por las trabajadoras sexuales en una agenda política que se opone a la lógica individualista, familiarista, doméstica y neoliberal del aislamiento, por medio de perspectivas comunitarias de cuidado, lo que se diseñó como la única línea de acción en salud para la categoría durante la pandemia. Las acciones colectivas reposicionan el trabajo sexual en la interfaz entre la salud pública y los derechos humanos y toman como principio el conocimiento de la calle, desde el activismo, y su poder de decisión sobre sus cuerpos.

## Introdução

Acompanhando os debates públicos suscitados por trabalhadoras sexuais ativistas e suas organizações durante o primeiro semestre de 2020, desenvolvemos o estudo *Eu Quero é Mais! A Vida de Profissionais do Sexo Durante a Pandemia da COVID-19*. A partir de um estudo de base comunitária, conduzido de forma remota, sob um olhar intersecional, nos propusemos conhecer os efeitos do que entendemos como marco pandêmico brasileiro, seus impactos e as respostas nacionais entre trabalhadoras sexuais (cis, trans e travestis) ativistas, lideranças comunitárias, dirigentes de organizações da sociedade civil (OSC) e trabalhadoras sexuais vivendo com HIV. A abordagem enfatiza particularidades e proximidades levando em consideração gênero, relações raciais, geração e âmbito de trabalho. O estudo não objetivou uma perspectiva comparada entre cidades.

A noção de marco pandêmico brasileiro nos ajuda a entender as vidas e relações sociais com e por meio da pandemia informados por perspectivas antropológicas ^1^ ^,^ ^2^ ^,^ ^3^. Aprofundar a leitura da pandemia enquanto um “fato social total” ^4^ implica considerar todo um enquadramento sociopolítico, econômico e material a partir do qual se produzem reelaborações com a presença virtual ou efetiva do vírus. Por essa perspectiva é relevante considerar os agenciamentos, tais como as estratégias institucionais para a COVID-19, as disposições institucionais e comunitárias de proteção de direitos, os interesses políticos em curso, os processos de produção de conhecimento, e as reelaborações, no geral, com implicações na vida das pessoas durante a pandemia.

Desde março de 2020, muito tem sido escrito sobre a pandemia de COVID-19 no Brasil e no mundo ^5^. No Brasil, a desastrosa resposta governamental federal foi criticamente descrita como uma “*estratégia de disseminação da COVID-19 no país*” ^6^. A forma como a pandemia de COVID-19 foi enfrentada produziu efeitos devastadores, acirrando desigualdades históricas, precarizando as condições de acesso à saúde pública e as condições materiais de existência e trabalho ^7^ ^,^ ^8^. Pesquisas que consideram o marco pandêmico têm mostrado como mulheres, pessoas negras, indígenas, LGBTQIA+ e classes populares sentiram em maior grau o impacto da pandemia, revelando o peso da determinação social da pandemia ^9^ ^,^ ^10^ ^,^ ^11^.

Para as trabalhadoras sexuais, o impacto não teria como ser diferente, tendo em vista o histórico de descaso da política brasileira para com a categoria ^12^. Nos primeiros meses de pandemia, as notícias expuseram a vulnerabilidade na qual viviam trabalhadoras sexuais de diferentes cidades a partir de um cenário de queda no número de clientes e programas, e insegurança sanitária e econômica. Em abril de 2020, ativistas e aliados organizaram diversas campanhas nas redes sociais (como Facebook e Whatsapp) para a arrecadação de dinheiro e donativos. Os laços institucionais, anteriormente estabelecidos com secretarias de saúde pública, secretarias da mulher e de direitos humanos, mostraram-se insuficientes. Nesse contexto, mensurar os impactos provocados pela pandemia de COVID-19 para trabalhadoras sexuais e demais populações vulnerabilizadas é uma tarefa complexa que vai exigir esforços de múltiplos estudos por algum tempo. Esperamos que este trabalho possa contribuir para os estudos do marco pandêmico brasileiro e para o campo da saúde pública, ao recuperar e atualizar o foco nas demandas políticas das trabalhadoras sexuais na intersecção entre a saúde e os direitos humanos.

A partir da análise das informações qualitativas coletadas na pesquisa *Eu Quero é Mais! A Vida de Profissionais do Sexo Durante a Pandemia da COVID-19*, buscamos compreender alguns dos impactos produzidos pela pandemia à saúde e vida de trabalhadoras sexuais entre 2020 e 2021. Para tal, dispusemos as análises em três eixos: (1) As várias faces da saúde e adoecimentos; (2) “Isolamento social”, práticas de prevenção e mutualidade de cuidados; e (3) Vacinação e estratégias coletivas.

## Metodologia

*Eu Quero é Mais! A Vida de Profissionais do Sexo Durante a Pandemia da COVID-19* fez parte da iniciativa EPIC, uma pesquisa multicêntrica de base comunitária implementada em 32 países sob coordenação da Coalizão PLUS (Coalition PLUS), uma rede global de organizações na luta contra o HIV e HCV. A iniciativa documentou o impacto da COVID-19 em populações historicamente afetadas pelo HIV e hepatite C, bem como em agentes comunitários de saúde ^13^.

No Brasil o estudo foi realizado entre os meses de julho e outubro de 2021, e composto por uma parte quantitativa e uma parte qualitativa, abrangendo trabalhadoras sexuais cis, trans e travestis de nove Unidades da Federação e 11 cidades diferentes: São Paulo e Campinas (São Paulo), Belo Horizonte e Uberlândia (Minas Gerais), Porto Alegre (Rio Grande do Sul), Belém (Pará), Brasília (Distrito Federal), Salvador (Bahia), Recife (Pernambuco), Natal (Rio Grande do Norte) e São Luís (Maranhão).

Participaram da pesquisa trabalhadoras sexuais cisgênero, lideranças do movimento brasileiro de prostitutas, vinculadas à Rede Brasileira de Prostitutas (RBP), à Articulação Nacional das Profissionais do Sexo (ANPROSEX) e à Central Única de Trabalhadoras e Trabalhadores Sexuais (CUTS), bem como integrantes do Coletivo/OSC Mulheres da Luz, da área do Parque da Luz, centro de São Paulo.

Entre as participantes trans e travestis, estiveram as ativistas que trabalham e residem na cidade de São Paulo e já participaram de pesquisas anteriores com a equipe do estudo, as trabalhadoras sexuais trans e travestis da região do Butantã, em São Paulo, e as da cidade de Uberlândia. É importante mencionar que boa parte da equipe de pesquisa tem uma trajetória de relações de pesquisa e aliança política com as trabalhadoras do sexo e suas organizações.

Ao longo do artigo, optamos por manter os nomes previamente autorizados de Lourdes Barreto, Fátima Medeiros e Cida Vieira, lideranças que participaram do estudo. Os outros nomes foram alterados para preservação das identidades.

Inicialmente realizamos um mapeamento das notícias publicadas na internet acerca das trabalhadoras sexuais no período e dos principais debates que circularam nos webinários em que participaram as organizações envolvidas. O mapeamento foi utilizado para formular questões para as entrevistas e orientar as interações em campo.

O componente qualitativo da pesquisa foi realizado de forma híbrida *online* e *offline*. Incluiu relatos etnográficos de trabalho de campo presencial, entrevistas em profundidade *online* e *offline*, e entrevistas *online*. Todas seguiram um roteiro semiestruturado, foram gravadas e transcritas. As entrevistas realizadas de forma remota encontraram obstáculos, tais como condição de acesso a recursos e equipamentos como *smartphone*, computador e rede de internet satisfatória. As atividades remotas não conseguiriam alcançar pessoas em situações de alta vulnerabilidade, como as que estão em abrigos ou em situação de rua.

Analiticamente, seguimos uma trilha interseccional ^14^ ^,^ ^15^, levando em consideração as formas de interação entre os marcadores sociais, o modo como produzem tensões no marco das desigualdades e diferenças. Marcadores de gênero, raça, classe e geração foram pensados em articulação a partir do contexto sociopolítico que particulariza o racismo e o sexismo no Brasil, e das condições que impõem ao trabalho sexual. Esses marcadores foram articulados com as dinâmicas próprias dos diversos âmbitos de trabalho sexual (particularidades locais, espaços mais fechados e mais abertos). O estudo não teve como objetivo realizar uma comparação entre cidades, mas identificar o que havia de comum e particular nas situações analisadas.

Nesse estudo, o marcador de geração, em relação a gênero, raça, classe e trajetórias de trabalho, resultou muito relevante. O estudo abrangeu trabalhadoras sexuais entre 22 e 80 anos e perpassou as categorias êmicas “mais novas” e “mais velhas”, “ativas” e “aposentadas”, tomadas a partir de uma diferenciação laboral. Entre as mulheres cis, os 50 anos é a idade indicativa de entrada no grupo das “mais velhas”, e entre as mulheres trans e travestis, essa transição ocorre por volta dos 40 anos, compreendendo contextos de risco e vulnerabilização potencializados por violências e transfobias ^16^, que tornam singulares as dinâmicas de envelhecimento desse grupo.

Ser “mais velha” remonta ainda à experiência de ativismo, de modo que, mesmo não estando mais “na ativa”, elas se reconhecem como trabalhadoras sexuais. Utilizamos a dimensão etária como um aspecto que permeia todo o estudo, considerando os sentidos geracionais expressos no campo de pesquisa e em articulação com os demais marcadores.

Em termos de verificação ética institucional, o estudo teve aprovação do Comitê de Ética em Pesquisa da Faculdade de Ciências Médicas da Santa Casa de São Paulo (CAAE: 39288920.5.0000.5479).

## Resultados e discussão

### As várias faces da saúde e do adoecimento

Das 43 entrevistadas na parte qualitativa, 14 relataram algum sintoma de “gripe” ou adoecimento em virtude da COVID-19 e 4 relataram “falta de ar”, principalmente as que já tinham diagnóstico prévio de doenças respiratórias. As mulheres trans e travestis de São Paulo e as trabalhadoras sexuais com mais idade foram as que mais citaram casos graves de adoecimento e mortes por COVID-19 entre familiares e clientes “mais velhos”. Sentimentos de pesar, medo e tristeza, seguidos da ênfase na defesa urgente da vacinação, marcaram as narrativas. A grande maioria não realizou testagem, embora tivessem pessoas próximas que se infectaram em processos oscilantes de adoecimento ou casos de falecimento.

As análises das trabalhadoras sexuais fizeram coro com as leituras sobre as necessidades impostas ao Sistema Único de Saúde (SUS) pela pandemia, considerando os desafios diante da gestão descentralizada, do subfinanciamento e da governança ^17^ ^,^ ^18^. A dificuldade de atendimento via SUS foi abordada pelas entrevistadas de Campinas, Salvador, Olinda, Natal e São Paulo, em especial por aquelas que acompanham o sistema de saúde há tempos, sobretudo enquanto ativistas com participação ativa nos projetos de prevenção de HIV e infecções sexualmente transmissíveis (IST). Entrevistadas relataram atrasos e falta de medicamentos durante a pandemia, redução dos insumos e das ações de prevenção comumente ofertados, como o oferecimento de testagens de IST e a distribuição de preservativos, além da ausência de repasse do lubrificante.

No quadro geral de adoecimentos, o efeito mais marcante nas entrevistas pode ser lido em termos de “saúde mental”, e está associado ao isolamento e à vulnerabilidade social e econômica. Pesquisas anteriores relataram em diferentes países a associação entre isolamento social e adoecimento mental como efeito determinante da pandemia de COVID-19 ^19^. A relação conflitiva entre a necessidade de trabalhar e o medo de morrer e de adoecer gravemente pode ser sintetizada na sentença: “*ou morro de COVID ou morro de fome*” ^20^. Depressão e ansiedade estiveram presentes em praticamente todas as narrativas. Também foram expressivas as falas acerca das dificuldades com o sono e do aumento do consumo de bebidas alcoólicas. Desespero e falta de expectativa de melhoria foram citados durante uma reflexão mais ampla sobre a crise sanitária em relação à falta de moradia, desemprego, insegurança alimentar, isolamento social, afastamento das redes de afeto e aumento da violência no marco do trabalho.

Nesse quadro, “ficar em casa” ocupa um lugar especialmente delicado. No rastro da literatura sobre prostituição no Brasil ^21^ ^,^ ^22^ ^,^ ^23^ ^,^ ^24^, a rua afirma-se como um espaço privilegiado de encontros e de relativa “liberdade”, enquanto “ficar em casa” significa isolar-se, flagrando-se como ameaça. Enquanto para mulheres negras e pobres, as vivências nas ruas sempre se deram de forma extensiva e relacionada à garantia de sustento ^25^, para a autoridade sanitária “ficar em casa” se constituiu como recomendação universal, atualizando o discurso moral e biopolítico do campo da saúde com seus conhecidos efeitos de disciplina ^26^.

Há consistentes narrativas de mulheres cis que expressaram angústias e violências nas relações familiares na quarentena, por exemplo aquelas que tinham o trabalho sexual como segredo ou que se sentiram mais ameaçadas no contato permanente com seus maridos. Esse achado vai ao encontro das pesquisas sobre violência doméstica e isolamento social ^27^. Destacamos a sensação de mal-estar descrita pelas jovens trans e travestis que tiveram que voltar e ficar em casas das quais se distanciaram, sendo obrigadas a manter um convívio com os familiares que não respeitam suas identidades de gênero.

Por outro lado, é importante destacar os casos das mulheres trans e travestis jovens, entre 24 e 30 anos, nordestinas e nortistas que tinham migrado recentemente e passaram a trabalhar na cidade de São Paulo, conforme observado na região do Butantã, e relataram o receio de ter que retornar às cidades de origem, nas quais estariam sujeitas a vivenciar novamente situações de transfobia ou desemprego.

Travestis e trans vivenciam uma situação paradoxal. Se por um lado o trabalho sexual nas ruas está associado ao risco de violências, por exemplo, já no primeiro ano de pandemia, em 2020, foi observado um aumento de 40% na violência e assassinato de pessoas trans no país, em relação ao ano anterior ^28^. A rua também pode ser vista como território de afeto e de cuidado, onde podem ser desejadas, vistas e reconhecidas em suas feminilidades múltiplas e divergentes.

Numa perspectiva que intersecciona a prostituição com geração e classe, as “mais velhas” vivenciaram períodos de extrema dificuldade em virtude das dinâmicas de distanciamento e afastamento das redes de afeto e da distribuição familiar dos trabalhos de cuidado, como se nota na entrevista com Lourdes Barreto (80 anos), uma das fundadoras da RBP e coordenadora do Grupo de Mulheres Prostitutas do Estado do Pará (GEMPAC): “*Essa crise sanitária é muito mais complicada, porque tu não pode beijar, tu não pode abraçar, tu não pode se aproximar, então é o momento mais difícil. Eu vivo todo dia assustada. Eu tô com traumas, transtorno mental, porque qualquer coisa eu me aborreço, choro também, porque tamo vivendo preso, sem poder sair, se sai é preocupada. Tamo vivendo o pior momento da história é agora. Nós tamos vivendo uma situação desesperadora da gente não conseguir dormir pensando se amanhã não é eu. Será que não é eu amanhã? Não é alguém que a gente conhece?*” (entrevista *online*, 9 de abril de 2021).

Em termos de saúde mental, o caso da Rua Guaicurus, em Belo Horizonte, se apresentou especialmente crítico como relatado por Cida Vieira: “*A gente tem pessoas que perderam os imóveis, tá virando moradoras de rua, tá na casa de parente, amigo, ou tá sofrendo alguma agressão. Ou teve que parar por tá grávida de parceiro mesmo, e aí a coisa se agrava. E tá unindo depressão e acaba entrando em dívida, a coisa não tá legal aqui em BH não. E tem o número de sífilis aumentado em Belo Horizonte entre as trabalhadoras sexuais e também as moradoras de rua*” (entrevista telefônica, 9 de abril de 2021).

Esse contexto de saúde mental motivou diversas organizações de trabalhadoras sexuais a promoverem atendimentos *online* individuais e em grupo, como a ANPROSEX e o Núcleo de Estudos da Prostituição (NEP), em Porto Alegre, as Tulipas do Cerrado, em Brasília, e a Associação das Prostitutas de Minas Gerais (APROSMIG), em Belo Horizonte. A procura por tratamento em saúde mental não é comum na trajetória do movimento de trabalhadoras sexuais, visto com desconfiança pelas recorrentes abordagens medicalizantes e moralizantes ^26^, e porque faz parte de uma seara entendida como “assistencialista”, que não obedeceria aos direcionamentos políticos do movimento. Entretanto, no marco pandêmico, essa se tornou uma ferramenta largamente demandada pelas trabalhadoras, transformando sagazmente o risco “assistencialista” em fortalecimento político.

### “Isolamento social”, práticas de prevenção e relações de cuidado

Práticas de “isolamento”, “quarentena” ou outras semelhantes não parecem ter sido, no campo do trabalho sexual, um recurso permanente e prioritário. Parar de trabalhar ou trabalhar em casa, de forma prolongada, não foram opções viáveis para a maioria das trabalhadoras sexuais. Experiências mais consistentes de isolamento estiveram presentes nos primeiros meses e semanas de circulação do vírus em cada local e, sobretudo, nas falas de mulheres cis “mais velhas” que tiveram suporte econômico de “clientes fixos”, amigos ou parentes.

Para mulheres trans e travestis o isolamento parece também ter sido difícil. Algumas das “mais jovens” e com programas mais lucrativos conseguiram deixar as ruas nos primeiros meses, o que se tornou possível devido ao uso de uma reserva financeira previamente constituída e às “ajudas” de clientes, mas voltaram ao trabalho quando não conseguiram mais se sustentar e perceberam que a pandemia teria longa duração. Essas chegaram a atender prioritariamente os clientes já conhecidos em suas próprias casas e algumas passaram a fazer atendimento por videochamada em seus celulares.

Enfrentando condições de precarização e vulnerabilidade, restrições de trabalho como forma de autocuidado foram abordadas em dimensões de mutualidade. Com clientes mais conhecidos, acordos de segurança foram feitos para a prevenção e diminuição do risco de contágio. Trabalhadoras que atendem clientes idosos relataram a preocupação de contaminá-los e, por vezes, recusaram-se a fazer o programa para não haver risco. De modo geral, os cuidados se revelaram mútuos e, como acontece nos tempos de “normalidade” ^29^, os clientes mais antigos figuraram como ajudas fundamentais.

Em termos de medidas de prevenção, o cotejamento do material de pesquisa sugere a prática de prevenção nos programas pela restrição da troca de saliva e do sexo oral, pela prática do sexo “de quatro” como estratégia para manter distância respiratória, e pelo uso da máscara.

Quanto ao uso da máscara, foi possível delinear três padrões distintos. O primeiro foi o uso consistente e atento ao tipo de máscara e à troca periódica, o que foi descrito como cotidiano pelas mulheres cis “mais velhas” e algumas trans, como foi possível constatar nas áreas de prostituição de Ceilândia (Brasília) e da região do Parque Jardim da Luz e da Estação da Luz (São Paulo).

O segundo foi de pouca adesão ao uso da máscara. Registramos o uso de máscaras de tecido e/ou muito largas, utilizadas por vários dias sem higienização, nariz para fora e máscaras cirúrgicas utilizadas repetidas vezes. Esse padrão foi observado no campo da pesquisa em São Paulo, Campinas e Brasília, e foi visto como comportamento habitual na prática geral de trabalhadores informais nessas cidades.

Com frequência, a máscara foi relacionada ao aumento do estigma. Nas palavras de Ariele, mulher trans que trabalha na região do Butantã, em São Paulo: “*então, quando a gente tava de máscara, os clientes ficavam: ‘ah será que já peguei? Será que aconteceu alguma coisa?’. Foi bem complicado. Aí tem que vir sem máscara*” (entrevista presencial, 20 de junho de 2021). Nas palavras de Fernanda, que trabalha na região do Butantã, o medo dos clientes remete também à ameaça social de mascarada como “marginal”: “*Já pensou a gente trabalhar: ‘ai, quanto é o programa?’. ‘É tanto’. ‘Com a máscara?’. O cliente quer ver, já tem aquela cisma de sair porque* [a gente] *é marginal. Vai sair mas ele tá arriscando a vida, aquela coisa com medo, porque os clientes sempre sai com a gente com medo*” (entrevista presencial, 20 de junho de 2021).

Assim, foi possível constatar que o não uso da máscara foi uma demanda masculina e um argumento de negociação para a realização ou não do programa, similar ao que ocorre com o preservativo ^30^.

O terceiro padrão revela usos informados e situados da máscara, que foram mudando no percurso dos meses, considerando as fases locais da pandemia, os aprendizados sobre o próprio vírus e suas circunstâncias de contágio. Por exemplo, por parte de algumas interlocutoras constatamos o não uso de máscaras em espaços abertos, sem aglomeração e sem evidência de sintomas, combinado com o uso restrito a espaços fechados como hotéis, escritórios, casas fechadas etc., o que se caracterizou como o mais adequado à medida que o conhecimento sobre os riscos de transmissão se ampliava. Destacam-se situações empiricamente “bem-sucedidas” (sem adoecimentos evidentes e com argumentação sobre a lógica de uso) de duas trabalhadoras e ativistas cis com grande atuação na redução de danos. É possível que a experiência com a redução de danos tenha guiado esse comportamento. Vale dizer que o uso de máscaras não tem sido objeto de estudos etnográficos nesse contexto.

Por fim, em termos de busca por medicamentos, remédios, “curas” e outras apostas terapêuticas, desenhou-se uma paisagem de navegação reflexiva por entre as controvérsias principais (cloroquina etc.), as possibilidades presentes no contexto e as necessidades de cada situação. A narrativa de Fátima Medeiros (Bahia) merece destaque pela forma como acionou os “medicamentos” naturais em detrimento dos receitados pelo posto de saúde e divulgados, sem embasamento científico, pelo Governo Federal.

“*Eu fiquei tão mal que eu não sabia o que fazer. E à noite era o pior momento, mas não tinha onde fazer um exame, e aonde eu ia não conseguia sequer ser atendida porque era gente demais. Na Ilha de Itaparica tem uns pés de eucalipto e comecei a tomar chá de eucalipto com mastruz, coisa da minha cabeça, se serviu eu não sei, mas eu fiz. Quando veio a segunda vez eu fiquei muito mal de novo. Passei no postinho no Pelourinho, fiz teste de COVID e deu positivo. Aí me passaram aquela rama de remédio amarguento, eu só tomei dois dias que eu não aguentei, e comecei a tomar os meus chazinhos à parte. Era tanta coisa que me deram. Me deram cloroquina, me deram ivermectina, me deram um bicho desse tamanho que eu não sei o nome, parecia um biscoito. Eu digo: ‘vou quebrar esse trem e vou comer, porque o bicho não vai passar aqui não’. Eu sei que o bicho amargava tanto e o coração fazia assim tum-tum. Não vou tomar senão eu vou morrer, esse trem vai acabar com meu coração*” (entrevista *online*, 15 de fevereiro de 2022).

### Vacinação e estratégias coletivas

Até o fim do período de levantamento de informações (outubro de 2021), todas as trabalhadoras sexuais com quem conversamos relataram ter tomado pelo menos uma dose da vacina e tinham expectativa de tomar as próximas. Algumas trabalhadoras trans e travestis que também são agentes de prevenção nos programas de saúde pública de São Paulo relataram ter se vacinado antes da agenda etária em virtude do trabalho realizado na área da saúde.

No movimento das trabalhadoras sexuais, houve uma mobilização consistente para conseguir acesso à vacinação de forma prioritária, sob a argumentação de que elas desempenhavam um trabalho essencial e deveriam ser consideradas profissionais de saúde, retomando o amplo histórico de atuação da categoria junto à saúde pública ^31^. Nem sob o argumento do trabalho essencial nem sob o espectro classicamente sanitarista da vulnerabilidade as trabalhadoras sexuais foram consideradas como prioritárias para a vacinação.

Organizações como Mulheres Guerreiras (Campinas), APROSMIG (Belo Horizonte) e o Coletivo/OSC Mulheres da Luz (São Paulo) acionaram estratégias capilares de gestão de xepas (vacinas não utilizadas no dia, que eram distribuídas para pessoas que estivessem em uma fila de espera) ou reservas de vacina para as trabalhadoras. Algumas estratégias merecem destaque, como as empregadas pela APROSMIG, que havia conseguido uma van para as ações de prevenção e passou a fazer rotas para a vacinação de trabalhadoras sexuais. A associação Mulheres Guerreiras, depois de intensa negociação com a unidade básica de saúde do território, conseguiu a vacinação das trabalhadoras do Jardim Itatinga e de outros trabalhadores do entorno com os quais se relacionam cotidianamente. Esse caso contou com a vacina Janssen, naquele momento prescrita em dose única, facilitando a imunização mais rápida e completa.

Em São Luís e Belém foram relatados raros casos de resistência à vacinação entre trabalhadoras sexuais cis por questões religiosas, influência de *fake news* e do discurso negacionista reproduzido maciçamente pelo Governo Federal acerca de supostos danos acarretados pelas vacinas.

Por fim, é importante anotar que, se não houve qualquer priorização em termos de testagem ou vacinação, no mesmo período houve a indicação prioritária das trabalhadoras sexuais para outro programa de saúde, situação que ajuda a traduzir bem a catástrofe político-sanitária brasileira. Em abril de 2021, o Ministério da Saúde, por meio da Secretaria de Ciência, Tecnologia, Inovação e Insumos Estratégicos em Saúde, publicou a *Portaria nº 13/2021* ^32^, que instituiu a oferta de um implante contraceptivo subdérmico para prevenção de gravidez não planejada especificamente a pessoas em “situação de rua”, vivendo com HIV/aids (em uso de dolutegravir e talidomida), privadas de liberdade e às trabalhadoras sexuais. A iniciativa causou imediata reação das trabalhadoras sexuais e demais lideranças dos grupos implicados que, por meio de cartas ao Ministério da Saúde, denunciaram-na como violação dos direitos sexuais e reprodutivos, e medida arbitrária de conotação discriminante e racista. Mobilizou também organizações civis e entidades de saúde pública, como a Associação Brasileira de Saúde Coletiva (ABRASCO), que publicou uma nota manifestando seu repúdio.

Diante dos novos desafios instaurados no marco pandêmico brasileiro, as organizações que atuam com trabalhadoras sexuais orientaram suas ações cotidianas para a arrecadação e distribuição de cestas básicas e *kits* de higiene, o compartilhamento de informações sobre prevenção em COVID-19, a continuidade do trabalho de prevenção em HIV e outras IST, a realização de acolhimentos afetivos, e a disponibilização de assistência terapêutica e outros cuidados em saúde. Todo esse trabalho foi realizado de forma *online* e *offline*, em redes locais e nacionais, mobilizando alianças de diversas naturezas, segundo as possibilidades de cada local.

## Considerações finais

O marco pandêmico brasileiro aprofundou de forma diferencial as condições de vulnerabilidade no cotidiano das trabalhadoras sexuais. A impossibilidade de adesão ao isolamento social e ao uso recomendado de máscaras tornou a situação ainda mais dramática. É a partir da ação e inação dos poderes públicos, sobretudo em relação a projetos e programas no âmbito da segurança do trabalho, da saúde pública e da promoção de direitos, que se pode ler a presença do vírus produzindo zonas limites de existência, em termos de fome e insegurança alimentar, falta de moradia, adoecimentos físico e mental, risco de infecção, dificuldade financeira, violência e precariedade no trabalho, reafirmando a determinação social da saúde ^33^.

Utilizando uma análise interseccional, foi possível compreender as particularidades do impacto na relação com gênero, raça, geração e âmbito de trabalho. Para a maioria das participantes da pesquisa, autodeclaradas pardas e pretas, as dificuldades se acirraram nos dois primeiros anos pandêmicos, suscitando intensos debates sobre racismo e dinâmicas raciais nos espaços de prostituição. No debate geracional, foi possível reconhecer as vulnerabilizações vividas, sobretudo, pelas “mais velhas”, cis, trans e travestis, quanto ao risco de infecção, adoecimento e desvalorização do valor do programa. As trabalhadoras cis falaram de como a dificuldade econômica afetou não só elas próprias, mas todo o sustento de familiares - mães, filhos e netos. Entre as trabalhadoras trans e travestis, foi relatado um aumento da discriminação no contexto de trabalho, relacionado ao uso da máscara e ao confronto com situações de transfobia para as que precisaram retornar à casa de familiares. Essa perspectiva endossa a leitura das principais pesquisas a respeito do marco pandêmico brasileiro como um contexto de acirramento das desigualdades estruturais.

É importante notar que nem nos dados quantitativos (não apresentados aqui), nem nos qualitativos, se evidencia a sensação de um processo de dizimação da categoria. A pesquisa não foi marcada por narrativas de mortes massivas ou sequenciais, apesar de que óbitos de pessoas mais ou menos próximas relacionados à COVID-19 estão presentes em todas as entrevistas, bem como nas redes pessoais da equipe de pesquisa. O que marca a análise é, de um lado, a sensação compartilhada acerca da existência de um profundo descaso social e político (incluindo a estratégia do Executivo Federal, mas também o SUS e suas limitações) com as vidas das trabalhadoras sexuais, e, do outro, a fragilização significativa da saúde mental e precariedade na vida e trabalho.

Diante do contexto crítico, as trabalhadoras sexuais ativistas tiveram de contar com suas próprias alianças para realizar ações de cuidado, assistência e prevenção nos territórios. Sobretudo as lideranças “mais velhas”, que, com mais trajetória no ativismo, desempenharam uma atuação fundamental na linha de frente da saúde pública, distribuindo máscaras e cestas básicas para as mais vulnerabilizadas ou doentes. Mesmo diante do profundo impacto, as ativistas não receberam apoio direto do Estado, num contexto de falência governamental nos territórios, recusa ao reconhecimento do trabalho sexual como um “trabalho essencial” e/ou inclusão da categoria no “grupo prioritário” de vacinação. Enfrentaram a dificuldade de acesso aos serviços públicos durante a pandemia, discriminações nos atendimentos médicos no SUS e casos de prescrição medicamentosa sem comprovação científica. Além do mais, precisaram responder à iniciativa moralizante no campo das políticas de saúde sexual e controle da reprodução, como a proposta de implantação do anticonceptivo subdérmico.

As reflexões das trabalhadoras sexuais endossam os principais debates mobilizados há décadas pelo movimento de prostitutas no questionamento da abordagem estigmatizante da prostituição no âmbito da saúde pública brasileira. Como princípio de ação, as trabalhadoras sexuais retomaram o histórico de cooperação entre a categoria e o Governo Federal nos trabalhos de prevenção em aids iniciados no final dos anos 1980, quando tomaram o protagonismo da pauta sob uma abordagem participativa e desenharam uma política em saúde mais aliada ao campo protetivo dos direitos humanos.

O conhecimento das ativistas em saúde no marco pandêmico pode ser lido a partir de uma agenda política pautada pelo apoio emocional, pela mutualidade de cuidados e pela ajuda assistencialista, contextual e emergencialmente acionada, envolvendo trabalhadoras sexuais cis, trans e travestis, familiares, clientes e outros personagens do entorno da prostituição. As realidades territoriais implicaram a produção de caminhos criativos de prevenção, apropriação das diretrizes sanitárias, uso de máscara e distanciamento em políticas circunstanciais de redução de danos. Resulta fundamental destacar que foi visível na pesquisa o desenvolvimento de diversas estratégias e pragmáticas de cuidado não coordenadas e não mediadas por nenhum agente centralizador, focadas numa lógica de redes e de mutualidade. Isto é, no cuidado de si, das famílias, dos clientes, dos entornos afetivos e econômicos, das agendas políticas e alianças no ativismo, em uma aposta na afirmação da vida, em contraposição à lógica individualista, familiarista e liberal de isolamento.

Mesmo com o fim da pandemia, os danos causados ainda marcam os territórios de prostituição, demandando ações continuadas nas organizações de trabalhadoras sexuais. As disputas políticas mantêm o enfoque no direito à vida, no reconhecimento do trabalho sexual e nas discussões em torno da saúde pública, questionando as disposições políticas em que estão implicadas somente como alvo de problemas sociais. Reclamam um reposicionamento que articule prostituição, saúde e direitos humanos, tomando como princípio o poder de decisão sobre seus corpos, a preservação da vida e as relações sociais desde as bases coletivas de ação.
